# Influence of lower anterior crowding on the predictability of mandibular tooth movement in Invisalign therapy: a retrospective cohort analysis

**DOI:** 10.3389/fdmed.2025.1727909

**Published:** 2026-02-06

**Authors:** Reem Y. Alulyan, Abdulaziz S. Alamri, Suliman Y. Shahin, Naif N. Almasoud, Essam A. Nassar, Osama A. Alsulaiman, Ahmed A. Alsulaiman

**Affiliations:** 1 College of Dentistry, Imam Abdulrahman Bin Faisal University, Dammam, Saudi Arabia; 2 Department of Preventive Dental Sciences, College of Dentistry, Imam Abdulrahman Bin Faisal University, Dammam, Saudi Arabia

**Keywords:** clear aligners, dental crowding, orthodontic appliances, orthodontic tooth movement, treatment outcome

## Abstract

**Introduction:**

Invisalign® can resolve up to 91.4% of mandibular incisor crowding. However, there is insufficient evidence to support clear aligner therapys effectiveness for various tooth movements. While the accuracy of Invisalign® has been evaluated, few studies have evaluated its efficacy in alleviating lower arch crowding. Hence, we aimed to identify significant differences between predicted and achieved linear, vertical, and rotational tooth movements in Class I mild-to-severe crowding cases. We also examined the association between lower anterior crowding and discrepancies in predicted vs. achieved movements.

**Methods:**

This retrospective cohort study, conducted in Saudi Arabia, included 44 participants collected between May and September 2023. The treatment protocol involved proclination followed by interproximal reduction for lower mandibular crowding.

**Results:**

The sample comprised 63% female patients (mean age: 32). All anterior lower teeth showed significant discrepancies between predicted and achieved movement (3.64 ± 3.25). A significant positive relationship was observed between crowding and lower right canine linear/rotational movements, as well as lower left central/lateral linear movements,lower left lateral vertical and lower right lateral linear movement. Clinically, a one unit increase in crowding correlated with a 0.49 increase in lower right canine rotational difference, while a one unit increase in crowding corresponded to a 0.405 increase in lower right lateral rotational difference.

**Conclusion:**

Dental crowding reduces the accuracy of clear aligner therapy. Clinically, overcorrection should be routine, and additional tools should be used to improve predictability in cases with significant crowding.

## Introduction

The concept of moving teeth using sequential removable appliances was first introduced by Kesling in 1945 (3). Align technology launched its clear aligner system, Invisalign, in 1999. Invisalign aligners move teeth approximately 0.33 mm every 14 days (4). A key feature of Invisalign treatment is the digital diagnosis and analysis of orthodontic cases using Clincheck software (5). Ideally, the initial treatment prediction should match the final clinical outcome to achieve optimal outcomes (6). Patient compliance is critical, as patients must wear the aligners for 20–22 h daily (7). A survey of orthodontists that looked at clear aligner practices revealed that 93.13% provide clear aligner therapy, which accounts for 24% of their annual income (8).

Anterior crowding is one of the main reasons patients seek orthodontic treatment (9). According to Buschang, 40% of the American population has unacceptable incisor irregularity, with 17% classified as severe (10). The need for orthodontic therapy as well as the prevalence of malocclusion was also assessed in Saudi Arabia, with 45.4% requiring orthodontic treatment, and crowding being the most frequent occlusal issue (11). The irregularity index, introduced by Robert Little, is a common method to assess lower arch anterior irregularity. This quantitative measure calculates the linear displacement between contact points of the mandibular incisors and canines, with the total of these measurements representing the Irregularity Index value (12). Another method evaluates crowding by comparing the mesiodistal diameter of a tooth to the available arch space, measuring crowding as the lack of space for a tooth in the dental arch (13).

Invisalign® offers an aesthetic advantage over fixed orthodontic treatment, making it more appealing to adults, with majority of the patients being above 19 years old (9). However, a study compared the treatment results of patients treated with Invisalign with patients treated with fixed conventional orthodontic appliances using the American Board Objective (ABO) grading system. The study found that clear aligner scores were significantly lower in improving overjet, posterior torque, antero-posterior occlusal relationships, and occlusal contacts (14). Another study investigated post-retention dental changes following Invisalign® and fixed conventional orthodontic appliances. Patients treated with Invisalign® experienced more relapse compared with those treated with fixed orthodontic appliances, particularly in the maxillary region (15). Jaber et al. found that patinets treated with Invisalign have less effect on on oral health-related quality of life when compared to patients treated with fixed orthodontic appliances (16). A systematic review comparing the treatment effectiveness of clear aligners compared to fixed orthodontic appliance found that clear aligners were associated with greater differences in predicted and achieved tooth movements compared to fixed orthodontic treatment. It also found that treatment using fixed orthodontic treatment resulted in better occlusal contact, buccalingualin inclination and shorter treatment duration than when compared to Invisalign treatment (17). Although fixed orthodontic appliances and Invisalign exhibit comparable overall efficacy, the duration of treatment with Invisalign is generally shorter (18).

Krieger et al. found that Invisalign® treatment outcomes resolve mandibular crowding through a combination of incisor proclination and interproximal reduction (6). Another study reported that approximately 91.4% of reduction of mandibular incisor crowding can be resolved by the Invisalign® treatment (1). However, previous studies stated that 80% of Invisalign® patients require case refinement before treatment completion (19, 20). Another study assessed the predictability of anterior teeth movements by comparing the Clincheck® vs. clinical treatment outcome. Multiple teeth movements were studied and the study reported that the anterior teeth achieved a mean accuracy of 41% using Invisalign® (21). Furthermore, a 2015 systematic review looked at the effectiveness of clear aligners in various tooth movements and found that the available literature was inconclusive, limiting evidence-based clinical decision (2).

Many studies have used various tools to assess Invisalign® accuracy. The Peer Assessment Rating scores and the ABO grading system have both been employed for this purpose (1). Buschang et al. compared predicted models with achieved outcomes using the ABO grading system and found that the predicted models did not resemble the clinical outcomes (22). Using printed models to produce clear aligners does not fully ensure that the resulting plates will fit optimally on the dental arches (23). Posttreatment models showed greater deductions than the ClinCheck® models, particularly in buccolingual inclinations, tooth alignment, occlusal relations, and occlusal contacts (22). Another method to assess Invisalign® accuracy is 3D superimposition of the predicted model and achieved models. A study examining specific tooth movements found that lower anterior intrusion was imprecise, although no significant differences were detected in horizontal movements (24). In 2020, Haouili reported Invisalign's® mean overall accuracy at 50%, with rotational movements showing the lowest accuracy at 46% (25). Grunheid et al. (2017) found that by superimposing the mandibular canine, lateral incisor, and first premolar rotations, mandibular incisor intrusion, and molar torque were the most difficult movements to achieve, with lower anterior movements showing very poor accuracy (26). Krieger et al. compared predicted and achieved models in 50 patients, noting a 0.4 mm difference in arch width and length (6). Limited data exists on Invisalign's® accuracy in alleviating moderate mandibular crowding, and most of the available information is based on clinical experience rather than evidence-based research. A well-conducted study of this kind may offer insightful data on clear aligner therapy. In this study, we aimed to assess Invisalign's® accuracy in achieving predicted tooth positions relative to the severity of anterior mandibular crowding and different types of tooth movements. The null hypothesis was that there would be no differences between the achieved and predicted tooth movements.

## Materials and methods

This retrospective cohort study was conducted in Saudi Arabia and included 44 participants collected between May 2023 and September 2023 with treatment initiation in 2019 or later. The sample size was calculated using G*Power 3, a statistical software tool for power analysis. For the paired t-test, the calculation was based on the following parameters: effect size (dz) of 0.5, 80% power, and an alpha level of 0.05 (24). The study received approval from the Institutional Review Board of Imam Abdulrahman Bin Faisal University (Ethics approval No. IRB-PGS-2023-02-419). Invisalign treatment was provided by multiple orthodontists in the university's orthodontic department, all following the same protocol for addressing lower anterior crowding. The protocol for treating lower mandibular crowding involved proclination followed by interproximal reduction, standard staging, no power ridges were used, and a similar attachment distribution. All subjects underwent a non-extraction treatment plan. Orthodontists planned Clincheck® using clincheck pro5 or pro6 versions and aligners were changed weekly. The clincheck Patients were directed to wear their aligners for 20–22 h daily and replace them every 7 days.

### Inclusion criteria

- Patients treated exclusively with Invisalign
- Patients in permanent dentition
- Tooth size-arch length discrepancy (crowding) in the lower anterior region exceeding 1 mm
- Treatment provided for both arches
- Completed initial and final intraoral scans
- Treatment began in 2014 or later, after Invisalign® introduced SmartTrack material®.

### Exclusion criteria

- Missing any lower anterior tooth
- Missing second molars.
- Patients with systemic diseases affecting tooth movement
- Patients taking medications that alter tooth movement.

After searching the provider orthodontist's Invisalign® database, patients meeting the criteria were selected, and their digital models were exported as STL files. The accuracy and reliability of 3D intraoral scanning methods are high compared with the gold-standard plaster models, and the observed differences were minimal and clinically insignificant (27). Full records were obtained for each patient, including demographics and three models: the initial scan, the predicted model (based on Clincheck®-planned movements), and the achieved model (final scan). Interproximal reduction (IPR) was recorded using the planned values specified in each patient's ClinCheck® treatment plan. The amount and location of IPR were extracted from the digital setup and documented for analysis. Using Geomagic software dental crowding was quantified by Little's Irregularity Index (12). After that the initial and predicted models were superimposed, followed by superimposition of the initial and achieved models. Differences were then computed. The superimposition reference, as described by Charalampakis et al., was the second molars, which remained stable in Clincheck® (24). Linear, vertical and rotational measurements were obtained in accordance to the method described by by Charalampakis et al, summarized in Table 1 (24). The examiner was blinded to participants’ sex, age, and name to eliminate bias. Intra-rater reliability testing yielded a mean intraclass correlation coefficient value between 0.75 and 0.9, demonstrating good reliability. This study followed the STROBE guidelines for cohort studies.

**Table 1:** Definitions of measurements taken.

Measurement	Definition
Rotational measurements	Selecting the most mesial and distal points on the incisal tip, drawing a straight line between them, repeating the same process for the tooth after treatment, and then measuring the angle formed between the two lines.
Linear measurements	Each tooth's incisal edge was measured using a ruler, and the midpoint was identified as the halfway point along this length. The midpoint of the incisal tip in the occlusal view was then linked to the corresponding midpoint after treatment, and the distance between the two points represented the vertical measurement.
Vertical measurements	Each tooth's incisal edge was measured using a ruler, and the midpoint was identified as the halfway point along this length. The midpoint of the incisal tip in the frontal view was then linked to the corresponding midpoint after treatment, and the distance between the two points represented the vertical measurement.

### Statistical analysis

Descriptive analysis included frequencies, means, and standard deviations. Normality test was performed using the Shapiro–Wilk test and Kolmogorov–Smirnov tests. A paired *t*-test was used to compare predicted and achieved tooth movements (i.e., actual tooth movement). Pearson correlation was used to evaluate the relationship between crowding severity and the accuracy of tooth movement (calculated as achieved/predicted × 100). The association between crowding severity and actual tooth movement was analyzed using a linear regression model. All statistical analyses were performed using SAS 9.4, with an alpha level set at <0.05.

## Results

The study included 44 participants, comprising 16 males (36.36%) and 28 (63.63%) females. The mean treatment time was 8.07 ± 2.33, with a minimum of 5 and a maximum of 15 (*n* = 44). The mean value of IPR in cm was 1.55 ± 0.64, with values ranging from 0.30 to 3.00 (*n* = 44). Rectangular attachments (3 mm) were placed on all lower canines, and all other treatment variables were standardized and identical across all patients. Descriptive statistics for demographics are presented in Table 2, while predicted and achieved tooth movements are summarized in Table 3. Paired *t*-tests were conducted to assess the accuracy of tooth movements. The achieved vertical movement of the lower right canine (M = 0.945, SD = 0.809) was lower than the predicted score (M = 1.25, standard deviation SD = 0.956), and paired-samples t-test revealed that this difference was statistically significant (MD = 0.309, SD = 0.335). Similarly, the lower right canine linear movement revealed a significant difference (MD = 0.34, SD = 0.34), with achieved scores (M = 1.45, SD = 0.809) lower than predicted (M = 1.79, SD = 0.956). In addition, rotational movement of the lower right canine also revealed a significant difference (MD = 3.46, SD = 3.25), with achieved scores (M = 13.66, SD = 9.19) lower than predicted (M = 17.12, SD = 11.09). In summary, the lower right canine exhibited significant differences in all parameters of movement parameters.

**Table 2:** Sample demographics.

Category	%, mean SD
Sex	
Male (*n* = 16)	36.36%
Female (*n* = 28)	63.64%
Age continuous	32.045(8.918)
Irregularity (continuous)	6.59(2.78)

**Table 3:** Descriptive statistics of predicted and achieved tooth movements.

Movement		Predicted		Achieved		Mean difference (predicted-achieved)	Percentage accuracy (achieved/predicted) × 100	*P* value
	*N*	Mean	SD	Mean	SD			
Lower right canine vertical movement	44	1.25	0.956	0.945	0.809	0.309(0.335)	75.6%	<.0001*
Lower right canine linear movement	44	1.79	0.965	1.45	0.809	0.34(0.34)	81.01%	<.0001*
Lower right canine rotational movement	44	17.12	11.09	13.66	9.19	3.46(3.25)	79.79%	<.0001*
Lower left canine vertical movement	44	1.29	0.934	1.03	0.934	0.26(0.47)	79.84%	<.0001*
Lower left canine linear movement	44	1.81	1.09	1.400	0.77	0.41(0.61)	77.35%	<.0001*
Lower left canine rotational movement	44	14.43	12.52	12.03	11.73	2.40(2.71)	83.37%	<.0001*
Lower right lateral vertical movement	44	1.84	1.24	1.22	0.97	0.62(0.63)	66.30%	<.0001*
Lower right lateral linear movement	44	2.28	1.23	1.71	1.00	0.56(0.541)	75.1%	<.0001*
Lower right lateral rotational movement	44	10.84	6.80	8.10	5.96	2.74(3.13)	74.72%	<.0001*
Lower left lateral vertical movement	44	1.93	1.17	1.42	0.96	0.51(0.53)	73.58%	<.0001*
Lower left lateral linear movement	44	2.38	1.47	1.87	1.47	0.51(0.56)	78.57%	<.0001*
Lower left lateral rotational movement	44	12.49	8.14	9.69	7.12	2.8(3.26)	77.58%	<.0001*
Lower right central vertical movement	44	1.602	1.111	1.234	0.918	0.37(0.52)	77.03%	<.0001*
Lower right central linear movement	44	2.212	1.101	1.7	1.101	0.51(0.6)	76.85%	<.0001*
Lower right central rotational movement	44	10.41	8.11	8.39	7.13	2.02(2.03)	80.59%	<.0001*
Lower left central vertical movement	44	1.75	1.127	1.202	0.86	0.55(0.58)	68.69%	<.0001*
Lower left central linear movement	44	2.183	1.18	1.73	0.88	0.45(0.52)	79.25%	<.0001*
Lower left central rotational movement	44	10.24	7.10	8.14	6.76	2.1(1.9)	79.49%	<.0001*
Sum of the linear difference	44	12.65	6.059	9.9	4.70	2.79(2.28)	78.26%	0.0196
Sum of the vertical difference	44	9.66	5.86	7.05	4.84	2.61(2.17)	72.98%	0.0252
Sum of the rotational difference	44	75.54	31.63	60.02	28.93	15.52(8.61)	79.45%	0.0185

* Significant <0.05.

The lower left canine showed results similar to that of the right canine, with significant differences in all movement parameters. The achieved vertical movement (M = 1.03, SD = 0.934) was lower than the predicted movement (M = 1.29, SD = 0.934), with a mean difference (MD) of 0.26 (SD = 0.47). Similarly, the achieved linear movement (M = 1.40, SD = 0.77) was lower than the predicted (M = 1.81, SD = 1.09), and a mean difference of (MD = 0.41, SD = 0.61). The rotational movement also showed a significant difference, with an achieved score(M = 12.03, SD = 11.73), and a predicted score of (M = 14.43, SD = 12.52), with a mean difference of (MD = 2.40, SD = 2.71).

The observed score for the lower right lateral vertical movement (M = 1.22, SD = 0.97) was lower than the predicted score (M = 1.84, SD = 1.24), which was significant with a mean difference of (MD = 0.62, SD = 0.63). Furthermore, the observed scores of both the lower right lateral linear and rotational movement had significant differences. The achieved linear movement (M = 1.71, SD = 1.00) was also lower than the predicted (M = 2.28, SD = 1.23), with a significant mean difference of (MD = 0.56, SD = 0.541). Similarly, the achieved rotational movement (M = 8.10, SD = 5.96) was lower than the predicted (M = 10.84, SD = 6.80), with a significant difference (MD = 2.74, SD = 3.13).

The lower left lateral incisor movements also scored lower than the predicted values across all parameters. The achieved vertical movement (M = 1.42, SD = 0.96) was below the predicted score (M = 1.93, SD = 1.17), with a significant mean difference (MD = 0.51, SD = 0.53). Similarly, the achieved lower left linear movement (M = 1.87, SD = 1.47) was lower than the predicted (M = 2.38, SD = 1.47), and the mean difference (MD=0.51, SD=0.56) was statistically significant. Lastly, the rotational movement followed the same trend, with achieved scores (M = 9.69, SD = 7.12) lower than predicted (M = 12.49, SD = 8.14) and a significant mean difference (M = 2.8, SD = 3.26).

For the lower right and left central incisors, all movement parameters showed significant mean differences of (MD = 2.79, SD = 2.28), (MD = 2.61, SD = 2.17), and (MD = 15.52, SD = 8.61), respectively. The lower right central incisor had achieved vertical (MD = 1.23, SD = 0.92), linear (MD = 1.7, SD = 1.10), and rotational movements (MD = 8.39, SD = 7.13) lower than their predicted counterparts (MD = 1.60, SD = 1.11; MD = 2.21, SD = 1.10; MD = 10.41, SD = 8.11, respectively). Similarly, the lower left central incisor had achieved vertical (MD = 1.202, SD = 0.86), linear (MD = 1.73, SD = 0.88), and rotational movements (MD = 8.15, SD = 6.76) lower than predicted (MD = 1.75, SD = 1.13; MD = 2.18, SD = 1.18; MD = 10.24, SD = 7.10, respectively). The sum of the linear, vertical, and rotational differences was significant, with mean differences of (MD = 2.79, SD = 2.28), (MD = 2.61, SD = 2.17), and (MD = 15.52, SD = 8.61), respectively. The sum of the achieved linear differences (M = 9.9, SD = 4.70) was lower than the predicted scores (M = 12.65, SD = 6.06). The sum of the achieved vertical differences (M = 7.05, SD = 4.84) was lower than predicted (M = 9.66, SD = 5.86). The sum of the achieved rotational differences (M = 60.02, SD = 28.93) was also lower than predicted (M = 75.54, SD = 31.63).

Table 4 demonstrated the relationship between crowding and movements. A significant positive relationship was observed between lower right canine linear movements and crowding, in terms of difference (*r* = 0.46, *p*-value = 0.002). In addition, the lower right canine rotational movement showed a significant positive association with crowding for difference (*r* = 0.421, *p*-value = 0.005). The lower right lateral linear movement also demonstrated a positive association with crowding for difference (*r* = 0.572, *p*-value = <.0001). There was a significant positive relationship between lower left central linear movement and crowding for difference (*r* = 0.355, *p*-value = 0.018). Furthermore, a significant positive relationship was observed between lower left lateral linear movement and crowding for difference (*r* = 0.627, *p*-value = <0.0001). Also, there was a significant positive relationship between lower left lateral vertical movement and crowding for difference (*r* = 0.380, *p*-value = 0.011).

**Table 4:** The relationship between crowding and movements.

Difference	Crowding
	R value (*p* value)
Lower right canine linear movement	0.457(0.002)*
Lower right canine vertical movement	0.202(0.188)
Lower right canine rotational movement	0.421(0.005)*
Lower right lateral linear movement	0.572(<.0001)*
Lower right lateral vertical movement	0.384(0.101)
Lower right lateral rotational movement	0.361(0.162)
Lower right central linear movement	−0.008(0.96)
Lower right central vertical movement	0.0718(0.643)
Lower right central rotational movement	0.004(0.98)
Lower left central linear movement	0.355(0.018)*
Lower left central vertical movement	0.225(0.141)
Lower left central rotational movement	0.7(0.656)
Lower left lateral linear movement	0.627(<0.0001)*
Lower left lateral vertical movement	0.380(0.011)*
Lower left lateral rotational movement	0.228(0.136)
Lower left canine linear movement	0.249(0.104)
Lower left canine vertical movement	0.773(0.618)
Lower left canine rotational movement	−0.009(0.956)

* Significant <0.05.

Table 5 illustrated the association between crowding and tooth movements. Incisor crowding was significantly associated with the lower right canine linear difference [*β* = 0.056, 95% confidence interval (CI) = 0.022–0.89, *p*-value = 0.0018]. A one-unit increase in crowding was associated with a 0.056 unit increase in the lower right canine linear difference. Similarly, the lower right canine rotational difference was significantly associated with incisor crowding (*β* = 0.491, 95% CI = 0.161–0.821, *p*-value = 0.0045), with a one-unit increase in crowding significantly associated with a 0.49-unit increase in the lower right canine rotational difference. Indicating that greater crowding correlated with a 0.49-unit increase in the lower right canine rotational difference. For the lower right lateral incisor, the linear, vertical, and rotational differences were all significantly associated with incisor crowding (*β* = 0.111, 95% CI = 0.062–0.161, *p*-value = 0.0001), (*β* = 0.087, 95% CI = 0.021–0.15, *p*-value = 0.0101), (*β* = 0.405, 95% CI = 0.08–0.73, *p*-value = 0.0162), respectively. With each one unit increase in crowding was associated with a 0.111, 0.087, and 0.405 increase in the lower right lateral linear, vertical, and rotational differences, respectively. Similarly, the lower left lateral linear and vertical differences were significantly associated with incisor crowding (*β* = 0.132, 95% CI = 0.081–0.183, *p*-value = <0.0001) and (*β* = 0.073, 95% CI = 0.017–0.128, *p*-value = 0.0109), respectively. A one-unit increase in the crowding was associated with a 0.132-unit increase in lower left lateral linear difference, while a one-unit increase in the crowding was associated with a 0.073-unit increase in lower left lateral vertical difference. In contrast, the lower right central incisor linear, vertical, and rotational differences were not significantly associated with incisor crowding. However, the lower left central incisor linear difference showed a significant association (*β* = 0.066, 95% CI = 0.012–0.119, *p*-value = 0.0182), with a one-unit increase in crowding linked to a 0.066-unit increase in lower left central incisor linear difference.

**Table 5:** Association between crowding (continuous) and movements.

outcome	Crowding				
	Intercept	Beta estimate	SE	95% CI	*P*-value
Lower right canine linear difference	−0.0319	0.0559	0.0168	(0.022–0.089)	0.0018^a^
Lower right canine vertical difference	0.148	0.0243	0.0182	−0.124–0.0611)	0.1881
Lower right canine rotational difference	−0.223	0.4914	0.1636	(0.1613–0.8216)	0.0045^a^
Lower left canine linear difference	0.055	0.0543	0.0326	(−0.0115–0.1201)	0.1035
Lower left canine vertical difference	0.173	0.0130	0.0259	(−0.0392–0.0653)	0.6176
Lower left canine rotational difference	2.456	-0.0084	0.1502	(−0.3115–0.295)	0.956
Lower right lateral linear difference	−0.170	0.111	0.0246	(0.0615–0.1607)	<0.0001^a^
Lower right lateral vertical difference	0.044	0.0872	0.0323	(0.021–0.152)	0.0101^a^
Lower right lateral rotational difference	0.072	0.405	0.162	(0.079–0.732)	0.0162^a^
Lower left lateral linear difference	−0.360	0.132	0.025	(0.081–0.1827)	<0.0001^a^
Lower left lateral vertical difference	0.029	0.073	0.027	(0.017–0.128)	0.0109^a^
Lower left lateral rotational difference	1.041	0.267	0.176	(−0.087–0.622)	0.136
Lower right central linear difference	0.523	–0.0017	0.033	(−0.068-0.065)	0.959
Lower right central vertical difference	0.279	0.0135	0.0289	(−0.045–0.0719)	0.643
Lower right central rotational difference	1.995	0.0033	0.113	(–0.224–0.231)	0.977
Lower left central linear difference	0.0219	0.066	0.027	(0.012–0.119)	0.0182^a^
Lower left central vertical difference	0.046	0.047	0.031	(−0.0163–0.110)	0.1413
Lower left central rotational difference	1.793	0.0459	0.102	(−0.161–0.253)	0.660

Difference = intercept + (beta estimate x crowding amount).

a Equation: Yi = B0 + B1 Xi.

## Discussion

The findings support the hypothesis that dental crowding reduces the accuracy of clear aligner therapy. This study highlights the relationship between crowding, individual teeth, and specific movements, examining whether crowding has a negative or positive impact on specific types of tooth movements. The use of superimposition tools provided precise insights into the accuracy of clear aligner therapy, particularly for specific movements. Previous studies relied on tools like the ABO and Objective Grading System to assess clear aligner accuracy; however, these methods were less precise than the superimposition technique used in this study (28). Earlier research also employed various methods to evaluate crowding resolution, such as the Peer Assessment Rating index and the ABO grading system, although only a few recent studies have utilized superimposition (29). Most studies focused on whether clear aligner therapy could resolve lower arch crowding; however, they did not investigate the relationship between crowding and predictability of tooth movements (30). Only a few studies considered crowding as a factor affecting predictability; however, they focused mainly on overjet and overbite, rather than individual teeth and specific movements (1). This study used superimposition to comprehensively assess the accuracy of clear aligner therapy, examining different types of tooth movements in relation to lower anterior crowding. The study included 44 patients, with 63% of the sample being female and a mean age of 32. Given the retrospective observational design, these findings should be interpreted as associative rather than causal.

A key finding in our study was that all anterior lower teeth exhibited significant discrepancies between predicted and achieved movements. Consistent with previous studies, predicted and achieved tooth positions differed, with rounded teeth such as mandibular canines showing lower accuracy in rotational movements (26). A recent study found that discrepancies between predicted and achieved results ranged from 2 to 3 mm, which was clinically significant. Only a limited number of cases showed close predictive accuracy (within 0.5 mm) (31). In this study, the lower right canine demonstrated the largest difference, particularly in rotational movement (MD = 3.46, SD = 3.25). In contrast, the lower left canine vertical movement showed the smallest difference between predicted and achieved outcomes (MD = 0.26, SD = 0.47). A recurring finding in prior studies is that rotational movements, particularly in canines, are the least accurate (24, 25, 32). This is likely due to the canine's large root surface area and long roots, which may hinder the achievement of predicted movements (33). Recent studies have similarly reported discrepancies between predicted and achieved tooth movements using digital treatment planning, supporting cautious interpretation of aligner predictability (31).

In general, anterior teeth are known to have poorer accuracy compared to other teeth. Both Krieger et al. and Kravitz et al. found that incisors exhibited low accuracy in vertical movement, with only 44% of the predicted movement achieved. Kravitz et al. also noted that, among anterior teeth, incisors showed the least accuracy in vertical movements, while canines were least accurate in rotational movements (6, 21, 26). When comparing the sum differences in this study, the rotational differences showed the largest difference between predicted and achieved outcomes (MD = 15.52, SD = 8.61). Other studies have similarly reported significant differences in rotational and linear movements in lower canines, aligning with our findings (26).

This study also revealed a significant positive relationship between lower right canine linear and rotational movements and crowding. As crowding increased, the difference between the predicted and the achieved increased. Similarly, the lower right lateral linear was positively associated with crowding. The lower left central, the lower left lateral linear movements and the lower left lateral vertical movement also showed a positive association with crowding. As crowding increased, greater discrepancies between predicted and achieved tooth movements were observed.

A unique finding in our study is the measurable association between crowding and specific tooth movements. For example, a one unit increase in crowding led to a 0.056 increase in the lower right canine linear difference. The lower right canine rotational movement revealed a clinically relevant result, with a one unit increase in crowding corresponding to a 0.49 increase in rotational difference. Similarly, a one unit increase in crowding resulted in a 0.405 increase in the lower right lateral rotational difference.

A prospective study on clear aligner therapy accuracy reported an overall accuracy of 50%, with linear movements being the most accurate at 56% (25). Meade et al. found that predicted overjet was closer to the achieved outcome compared with overbite correction, suggesting that linear movements are easier to achieve than vertical movements (28). Some studies have found no significant differences in linear movements between predicted and achieved outcomes (24). However, the results of this study differ, as the sum of the linear differences between predicted and achieved movements was statistically significant.

The clinical implications of this study are significant. Since achieved outcomes are often less than predicted movements, overcorrection should be routinely incorporated. In cases with clinically relevant crowding, additional tools should be used to improve predictability. Clinicians should also be aware of the challenging movements in clear aligner therapy and incorporate necessary overcorrections to improve overall accuracy.

This study has some limitations. As a retrospective study, the risk of selection bias exists, although this was mitigated by a large sample size and strict inclusion/exclusion criteria. Furthermore, patient compliance was not assessed, which is a common limitation in retrospective studies. The superimposition method used the second molars as a reference, assuming they were stable; however, minor movements were detected. Some movements demonstrated relatively low accuracy, likely due to insufficient overcorrection. Notably, a 50% accuracy in predicted tooth movement does not necessarily equate to 50% clinical effectiveness, as overcorrection was not part of the treatment protocol for the sample.

## Conclusions

In conclusion, our findings revealed that:

1. Dental crowding reduces the accuracy of clear aligner therapy, particularly for specific tooth movements, with all anterior lower teeth showing significant discrepancies between predicted and achieved movements.
2. Rotational movements in mandibular canines were the least accurate, with the lower right canine showing the largest difference, while the lower left canine vertical movement had the smallest difference.
3. Rotational movements had the largest overall difference between predicted and achieved outcomes
4. A positive relationship was found between crowding and accuracy for lower right canine linear and rotational movements, lower right lateral linear and rotational movements, and lower left central and lateral linear movements.

These findings highlight the challenges of clear aligner therapy in cases of dental crowding and suggest the need for overcorrection and improved predictability in treatment planning.

Future studies should incorporate additional measurements, such as volumetric 3D cone-beam imaging, to evaluate the predictability of tooth movements and provide an accurate representation of root movements within the clear aligner system. This study was limited by a small sample size due to highly selective inclusion criteria. Future research should include more prospective studies and randomized trials with larger sample sizes to offer more generalizable findings on the clear aligner's predictability.

## Data Availability

The original contributions presented in the study are included in the article/Supplementary Material, further inquiries can be directed to the corresponding author.
